# Therapeutic regimen of l-arginine for MELAS: 9-year, prospective, multicenter, clinical research

**DOI:** 10.1007/s00415-018-9057-7

**Published:** 2018-09-29

**Authors:** Yasutoshi Koga, Nataliya Povalko, Eisuke Inoue, Hidefumi Nakamura, Akiko Ishii, Yasuhiro Suzuki, Makoto Yoneda, Fumio Kanda, Masaya Kubota, Hisashi Okada, Katsunori Fujii

**Affiliations:** 1 Department of Pediatrics and Child Health, Kurume University School of Medicine, 67 Asahi-machi, Kurume, Fukuoka 830-0001 Japan; 2 Institute of Fundamental Medicine and Biology, Open Lab Gene and Cell Technology, Kazan Federal University, Kazan, Russia; 3 Division of Medical Informatics, St. Marianna University School of Medicine, Kawasaki, Japan; 4 Center for Clinical Research and Development, National Center for Child Health and Development, Setagaya, Japan; 5 Department of Neurology, Tsukuba University School of Medicine, Tsukuba, Japan; 6 Department of Pediatric Neurology, Osaka Women’s and Children’s Hospital, Osaka, Japan; 7 Department of Neurology, Faculty of Nursing and Social Welfare Sciences, Fukui Prefectural University, Fukui, Japan; 8 Department of Neurology, Kobe University Hospital, Kobe, Japan; 9 Division of Neurology, National Center for Child Health and Development, Setagaya, Japan; 10 Department of Neurology, Nagoya Medical Center, Nagoya, Japan; 11 Department of Pediatrics, Chiba University Graduate School of Medicine, Chiba, Japan

**Keywords:** l-Arginine, Mitochondrial disease, MELAS, Stroke-like episodes, Ictus

## Abstract

**Objective:**

To examine the efficacy and safety of the therapeutic regimen using oral and intravenous l-arginine for pediatric and adult patients with mitochondrial myopathy, encephalopathy, lactic acidosis, and stroke-like episodes (MELAS).

**Methods:**

In the presence and absence of an ictus of stroke-like episodes within 6 h prior to efficacy assessment, we correspondingly conducted the systematic administration of oral and intravenous l-arginine to 15 and 10 patients with MELAS in two, 2-year, prospective, multicenter clinical trials at 10 medical institutions in Japan. Subsequently, patients were followed up for 7 years. The primary endpoint in the clinical trial of oral l-arginine was the MELAS scale, while that for intravenous l-arginine was the improvement rates of headache and nausea/vomiting at 2 h after completion of the initial intravenous administration. The relationships between the ictuses of stroke-like episodes and plasma arginine concentrations were examined.

**Results:**

Oral l-arginine extended the interictal phase (*p* = 0.0625) and decreased the incidence and severity of ictuses. Intravenous l-arginine improved the rates of four major symptoms—headache, nausea/vomiting, impaired consciousness, and visual disturbance. The maximal plasma arginine concentration was 167 μmol/L when an ictus developed. Neither death nor bedriddenness occurred during the 2-year clinical trials, and the latter did not develop during the 7-year follow-up despite the progressively neurodegenerative and eventually life-threatening nature of MELAS. No treatment-related adverse events occurred, and the formulations of l-arginine were well tolerated.

**Conclusions:**

The systematic administration of oral and intravenous l-arginine may be therapeutically beneficial and clinically useful for patients with MELAS.

## Introduction

Mitochondrial myopathy, encephalopathy, lactic acidosis, and stroke-like episodes (MELAS), a clinical phenotype of mitochondrial encephalomyopathies [1–3] and first reported by Pavlakis et al. [4], is a progressively neurodegenerative and eventually life-threatening mitochondrial disorder that causes anatomohistopathological and clinical findings (e.g., A3243G point mutation in mtDNA, ragged red fibers, and stroke-like episodes). MELAS has the estimated prevalence rate of 10–15 cases per 100,000 persons [5], and our 5-year, nationwide, multicenter, prospective cohort study in Japan [6] identified MELAS as the most common clinicopathologic type of mitochondrial disorders and indicated the following facts: the mortality rates from sudden death were 17.6% (3/17) and 33.3% (1/3) among 20 deaths in 58 patients with juvenile-onset MELAS (< 18 years) and 38 patients with adult-onset MELAS (≥ 18 years), respectively; and juvenile-onset MELAS was associated with a significantly higher mortality and more rapid disease progression than adult-onset MELAS, with a greater risk of renal failure an increasingly recognized clinical feature of mitochondrial cytopathies [7]. MELAS is distinguishably characterized by the sudden, transient, and recurrent development of stroke-resembling symptoms (headache, nausea/vomiting, visual disturbance/visual field abnormalities, seizures, and impaired consciousness: ictus), with the distribution of brain lesions that are incongruent to the usual vascular territories [8]. Furthermore, MELAS may manifest clinically in the interictal phase (ictus-free phase), “interictal MELAS” and in the acute phase (ictus-developing phase) “acute MELAS.”

l-Arginine therapy has been suggested to be beneficial for patients with MELAS [6, 9–15]. We had conducted a 6-month background survey on the interictal phases of candidate patients for our studies. The objective of the present clinical research was to examine the efficacy and safety of the therapeutic regimen using l-arginine for patients with MELAS.

## Methods

### Study design

The OL-MELAS Research is a 9-year clinical study that integrated the pooled data from two, 2-year, phase 3, prospective, multicenter, open-label clinical trials of oral and intravenous l-arginine in patients with juvenile- or adult-onset MELAS; the research was conducted at ten medical institutions in Japan in accordance with the Declaration of Helsinki and Good Clinical Practice guidelines. The protocols were approved by the institutional review board at each site. Oral l-arginine and intravenous l-arginine were purchased commercially (Ajinomoto Pharmaceutical Co., Ltd., Tokyo, Japan). The manufacturer was not involved in the conduct of the present study at all. All authors were involved in data interpretation and manuscript editing, vouched for the completeness and accuracy of the data, as well as for the compliance of the trials with the protocols, and agreed to submit the manuscript for publication. The clinical trials were registered at Center for Clinical Trials, Japan Medical Association (registry numbers: JMACTR-IIA00023 and JMACTR-IIA00025).

### Research population

Patients were eligible in the clinical trial of oral l-arginine if they developed stroke-like episodes in the last 2 years, had the A3243G point mutation in mtDNA, had never been treated with oral l-arginine, and clinical manifestations of stroke-like episodes were evaluable. In the clinical trial of intravenous l-arginine, eligible patients were diagnosed with MELAS, had the A3243G point mutation in mtDNA, and developed an ictus of stroke-like episodes within the previous 6 h.

**Fig. 1:**
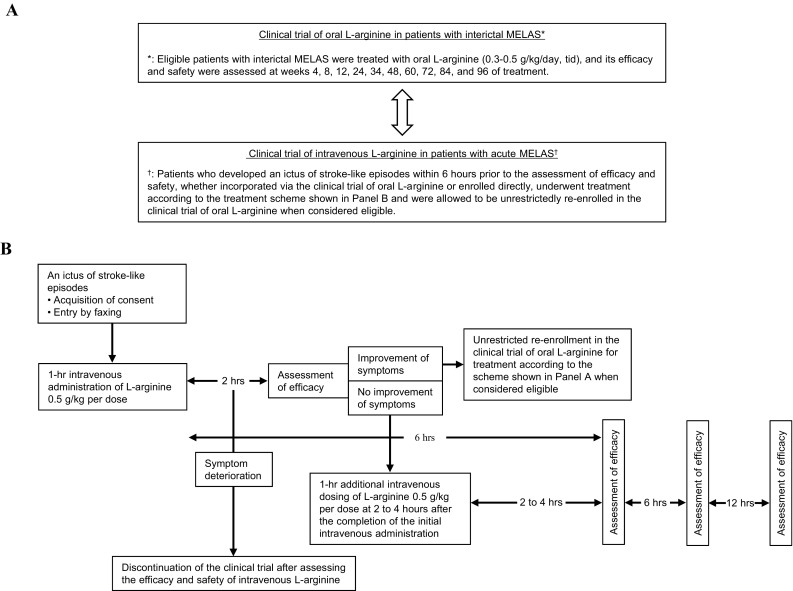
Therapeutic regimen using oral and intravenous l-arginine for patients with MELAS. **a** Diagram showing the bidirectionality of the therapeutic regimen using oral and intravenous l-arginine for patients with interictal or acute MELAS. **b** Diagram showing the scheme of pharmacotherapy with intravenous l-arginine for patients with acute MELAS. tid, ter in die; MELAS, mitochondrial myopathy, encephalopathy, lactic acidosis, and stroke-like episodes

Patients, who had been incorporated in the clinical trial of intravenous l-arginine from the clinical trial of oral l-arginine or enrolled directly into the former trial, were allowed to be bidirectionally and unrestrictedly re-enrolled in the clinical trial of oral l-arginine when considered eligible (Fig. 1).

### Research procedures

Plasma arginine concentrations need to be maintained to 100 µmol/L at minimum for the preservation of normal endothelial function in patients with MELAS [11]. Hence, patients received l-arginine 0.3–0.5 g/kg/day orally in three divided doses after each meal for 2 years. Patients who developed an ictus of stroke-like episodes received l-arginine 0.5 g/kg per dose intravenously, with the additional dosing of intravenous l-arginine 0.5 g/kg per dose at 2 h after completion of the initial intravenous administration when symptoms did not improve.

### Endpoints

In the clinical trial of oral MELAS, the primary endpoint for efficacy was the MELAS stroke scale; the secondary endpoints were “the mitochondrial disorder severity scores,” “time from oral l-arginine administration to the first ictus (time to the first ictus),” and “the migraine severity scores.” Six symptoms of stroke-like episodes [headache, nausea/vomiting, paresis of the extremities, seizures, impaired consciousness, and visual disturbance (teichopsia and cortical blindness)] were scored and integrated in 4-week units. To examine the ictus-preventing effect of oral l-arginine, physicians assessed the time to the first ictus by calculating the cumulative probability rate according to the Kaplan–Meier method. Using the migraine disability assessment score questionnaire, physicians calculated the migraine severity scores in units of 4 weeks.

In the clinical trial of intravenous l-arginine, the primary endpoints for efficacy were the improvement rates of headache and nausea/vomiting at 2 h after completion of the initial intravenous administration; the secondary endpoints were (1) improvement/no improvement in four major symptoms (headache, nausea/vomiting, impaired consciousness, and visual disturbance) as assessed based on physician’s determinations and changes in the visual analogue scale (VAS), (2) changes in each of these four major symptoms, and (3) overall assessment based on the physician’s subjective view (see the protocols). The safety of oral l-arginine and intravenous l-arginine was assessed based on adverse events (AEs), as well as changes in laboratory values and vital signs.

The Japanese mitochondrial disorder rating scale (JMDRS) (Table 1) were used to assess MELAS progression during the 2-year clinical trials and the subsequent 7-year follow-up, and the Newcastle Mitochondrial Disorder Adult Scale (NMDAS) [16] during the latter.

**Table 1:** JMDRS

Section 1: activities of daily living
A. Speech
0: Normal
1: Mildly affected, no difficulty being understood
2: Moderately affected, may be asked to repeat
3: Severely affected, frequently asked to repeat
4: Unintelligible most of time
B. Swallowing
0: Normal
1: Rare choking
2: Occasional choking
3: Requires soft food
4: Requires nasogastric or gastrostomy tube
C. Handwriting
0: Normal
1: Slightly small or slow
2: All words small but legible
3: Severely affected, not all words legible
4: Majority illegible
D. Cutting food- handling utensils
0: Normal
1: Somewhat slow and clumsy but no help needed
2: Can cut most foods, some help needed
3: Food must be cut but can feed self
4: Needs to be fed
E. Dressing
0: Normal
1: Somewhat slow and clumsy but no help needed
2: Occasional help with buttons or arms in sleeves
3: Considerable help required but can do some things alone
4: Helpless
F. Hygiene
0: Normal
1: Somewhat slow and clumsy but no help needed
2: Needs help with shower or bath or very slow in hygienic care
3: Requires assistance for washing, brushing teeth, going to bathroom
4: Helpless
G. Falling
0: None
1: Rare falling
2: Less than once per day
3: Average of once per day
4: More than once per day
H. Paroxysmal event (migraine, seizures)
0: None
1: < 1 every 1 month
2: > 1 every 1 month < 1 every week
3: > 1 every 1 week < 1 every day
4: > 1 every day/status
Section 2: motor
A. Proximal muscle strength (modified MRC)
0: Normal
1: Slight reduction of power (grade 4 MRC)
2: Moderate impairment, able to overcome gravity (MRC3)
3: Severe weakness, unable to overcome gravity (MRC2)
4: Severe weakness, flicker only (MRC1)
5: No voluntary muscle activity (MRC0)
B. Upper limb coordination (modified ICARS: International Cooperative Ataxia Rating Scale)
0: Normal
1: Mild clumsiness: no significant disability
2: Moderate clumsiness: poor writing, able to perform ADL
3: Severe clumsiness: unable to write
4: Severe clumsiness: unable to feed
C. Walking
0: No limitation
1: Mildly limited (gets tired after 1–2 km)
2: Moderately limited (difficulties keeping up with friends)
3: Severe limited (having to stop every 100–400 m to rest)
4: No walking distance beyond 10 m
D. Moderate motor activities (e.g., vacuum cleaning, carrying groceries, climbing one flight of stairs, preparing bed)
0: No limitation
1: Mildly limited
2: Moderately limited
3: Severely limited
4: Not capable
E. Vigorous motor activities (e.g., running, climbing several flights of stairs, or participating in other strenuous sports)
0: No limitation
1: Mildly limited
2: Moderately limited
3: Severely limited
4: Not capable
Section 3: special sensory
A. Vision
0: Normal
1: Unable to drive or equivalent (i.e., unable to read traffic or shop signs)
2: Unable to read normal print books
3: Unable to read standard large print books
4: Unable to watch TV
5: No useful vision
B. Auditory
0: < 10 dB loss
1: 10–20 dB loss
2: 20–40 dB loss
3: Severe > 40 dB loss but improves with hearing aid
4: Severe > 40 dB loss and does not improve with hearing aid
Section 4: endocrine
0: Normal
1: Single endocrine organ involvement
2: Two endocrine organs involved
3: Three endocrine organs involved
For diabetes, add 1 for insulin-treated
Section 5: cardiac
0: Normal ECG and ECHO
1: Conduction system disease, mild impaired LV function (EF > 60%) or asymptomatic hypertrophy
2: ECHO evidence of cardiomyopathy and restricted physical activity (EF < 60%) or cardiac pacemaker, moderate cardiomyopathy (EF < 40–60%)
3: Severe cardiomyopathy
Section 6: renal
0: Normal
1: Creatinine clearance < 50–90%
2: Creatinine clearance 30–50%
3: Creatinine clearance 10–30%
4: Creatinine clearance < 10 ml/min or transfusion
Section 7: cognition and impairment
A. Intellectual impairment
0: Normal
1: Mild (consistent forgetfulness with partial recollection of events but no other difficulties)
2: Moderate memory loss with disorientation and moderate difficulty handing complex problems
3: Severe memory loss with disorientation as to time and often place, severe impairment with problems
4: Severe memory loss with orientation only to person, unable to make judgments or solve problems
B. Motivation and drive
0: Normal
1: Lacking in energy, activities not restricted
2: Lacking in energy, restricts hobbies and interests
3: Lacking in energy, restricts day-to-day (routine) activities
4: Unable to carry out any task

JMDRS is modified from the European NeuroMuscular Conference (ENMC) mitochondrial disease rating scale published in Neuromuscular Disorders. 2003;13(9):757–764

*JMDRS* Japanese mitochondrial disease rating scale

### Plasma arginine concentrations

In the clinical trial of oral l-arginine, plasma arginine concentration were measured at 2 h after ingestion, and the dose was adjusted in the dose range of 0.026–0.5 g/kg/day as granules to allow the sustainment of plasma arginine concentrations of 100 µmol/L at minimum at weeks 4, 8, 12, 24, 36, 48, 60, 72, 84, and 96. In the clinical trial of intravenous l-arginine, plasma arginine concentrations were measured before the first intravenous administration at the hospital and at 24 h thereafter.

### Statistical analysis

For the efficacy analysis, the primary analysis set was the full analysis set. Safety was analyzed based on the safety analysis set that consisted of patients who received at least one dose of the investigational drug. Descriptive statistics (e.g., mean, standard deviation, counts, and proportions) were summarized as appropriate. The primary endpoint was analyzed by one-sample Wilcoxon’s signed-rank test. The 9-year survival of patients was calculated according to the Kaplan–Meier method. The mitochondrial disorder severity scores and the migraine severity scores were analyzed by one-sample Wilcoxon’s signed-rank test. The improvement rates of headache and nausea/vomiting at 2 h after completion of the initial intravenous administration were calculated separately and analyzed by Fisher’s exact test. The multiplicity issue was adjusted according to the Hochberg procedure [17]. One-tailed t-test was conducted to examine the between-group difference with respect to the following two interictal phases: one between the day of first diagnosis and the day of first ictus; and another between 60 days prior to the day of first diagnosis and the day of first ictus. A value of *p* < 0.05 (one-tailed) was considered statistically significant. All statistical analyses were made with the SAS software package version 9.2 (SAS Institute, Cary, NC).

## Results

From March 2009 through June 2011, 15 among 96 patients who had been identified in our cohort study [6] were enrolled in the clinical trial of oral l-arginine; three discontinued trial treatment due to the increased frequency of epileptic seizures, concurrent pneumonia, and unverified efficacy, respectively. Consequently, 12 completed the trial. The efficacy of oral l-arginine was assessed for 13 patients who were alive but had no data on NMDAS, and the safety of the drug for 15 patients. From December 2008 through March 2011, 10 patients were enrolled in the clinical trial of intravenous l-arginine; no treatment discontinuation occurred. Hence, ten completed the trial. The efficacy and safety of intravenous l-arginine were analyzed for ten patients each. Five patients were enrolled in both clinical trials. Three patients received intravenous l-arginine in seven episodes. Two patients with adult-onset MELAS one patient each with interictal and acute MELAS were lost to follow up due to the transfer of the investigator; they are alive but unevaluable for the JMDRS or NMDAS scores. Consequently, 18 and 20 patients were analyzed for the efficacy and safety of the therapeutic regimen using oral or intravenous l-arginine, respectively.

No patient died or became bedridden at the completion of the 2-year clinical trial, and 13 patients were followed up until May 31, 2017. At the completion of the 7-year follow-up, none of them became bedridden; the numbers of patients with interictal and acute MELAS were 11 and 8, respectively, because 4 deaths had occurred 1 each from renal failure and sudden death with respect to interictal MELAS, as well as 1 each from sudden death and renal and heart failure with respect to acute MELAS. During the 7-year follow-up, all patients who had completed the 2-year clinical trial of intravenous l-arginine received the drug when developing an ictus of stroke-like episodes, but not all of them received oral l-arginine during the interictal phase; on the other hand, all of patients who had completed the 2-year clinical trial of oral l-arginine continued receiving oral l-arginine during the interictal phase and received intravenous l-arginine when developing an ictus of stroke-like episodes. Patient characteristics at baseline, at the completion of the 2-year clinical trials of oral and intravenous l-arginine, and at the completion of the 7-year follow-up are shown in Tables 2, 3, and 4, respectively. No ictus developed in five patients with interictal MELAS (38.4%), while four (30.8%) experienced three or more ictuses during the clinical trial of oral l-arginine. In the subpopulations of patients with interictal or acute MELAS, time from diagnosis to the first enrollment/incorporation was longer for juvenile-onset MELAS than for adult-onset MELAS (Table 2). Five patients each were enrolled in the clinical trials of oral and intravenous l-arginine, and four patients were enrolled in the clinical trial of intravenous l-arginine only. Four patients, who had taken l-arginine other than the study drug prior to the onset of the present study, were enrolled in the clinical trial of intravenous l-arginine only (Table 3).

**Table 2:** Characteristics of pediatric and adult patients with interictal or acute MELAS at the baseline of the 2-year clinical trials of oral and intravenous l-arginine

Characteristics	Interictal MELAS (*N* = 13)^a^	Acute MELAS (*N* = 10)^a,b^
Sex		
Male, no. of patients (%)	7 (53.8)	8 (80.0)
Age, years, no. of patients (%)		
< 18	6 (46.2)	5 (50.0)
≥ 18	7 (53.8)	5 (50.0)
Mean ± SD	20.7 ± 12.5	17.2 ± 5.1
Onset type of MELAS, no. of patients (%)		
Juvenile-onset (< 18 yrs)	8 (61.5)	8 (80.0)
Time from diagnosis to the first enrollment/ incorporation, years		
Juvenile-onset (< 18 years)	1.75 ± 1.67	3.63 ± 3.07
Adult-onset (≥ 18 years)	0.80 ± 1.10	2.0 ± 1.41
All onset types	1.38 ± 1.50	3.3 ± 2.83
Number of ictuses that occurred in the last 1 year, no. of patients (%)		
0	5 (38.5)	NA
1	5 (38.5)	NA
2	0 (0.0)	NA
3	1 (7.1)	NA
4 or more	2 (85.8)	NA
Unknown (< 1-year monitoring)	0 (0.0)	NA
All frequency ranges	13 (100.0)	
Number of anticonvulsants used previously—no. of patients (%)		
1	8 (61.5)	2 (20)
2	3 (23.1)	4 (40)
≥ 3	2 (15.4)	4 (40)
Duration of treatment with the first anticonvulsant, months		
Mean ± SD	2.29 ± 2.25	3.40 ± 2.67
Plasma arginine concentration, µmol/L		
Mean ± SD	65.74 ± 16.74	NA
JMDRS—scores in sections 1 and 2^c^		
0–10: no. of patients (%)	10 (76.9)	5 (50.0)
11–20: no. of patients (%)	2 (15.4)	3 (30.0)
21–30: no. of patients (%)	1 (7.7)	2 (20.0)
≥ 31: no. of patients (%)	0 (0.0)	0 (0.0)
All score ranges, mean ± SD	7.29 ± 8.77	11.1 ± 10.2

Values are expressed mean ±  SD

*MELAS* mitochondrial myopathy, encephalopathy, lactic acidosis, and stroke-like episodes, *SD* standard deviation, *NA* not applicable, *JMDRS* Japanese mitochondrial disease rating scale

^a^Five patients each were enrolled in the clinical trials of oral and intravenous l-arginine. *N* represents the full analysis set

^b^Four patients, who had taken l-arginine other than the study drug prior to the onset of the present study, were enrolled in the clinical trial of intravenous l-arginine only

^c^Score range: 0–81

**Table 3:** Characteristics of pediatric and adult patients with interictal or acute MELAS at the end of the 2-year clinical trials of oral and intravenous l-arginine

Characteristics	Interictal MELAS (*N* = 13)^a^	Acute MELAS (*N* = 10)^a,b^
Sex		
Male, no. of patients (%)	7 (53.8)	8 (80.0)
Age, years, no. of patients (%)		
< 18	5 (38.5)	5 (50.0)
≥ 18	8 (61.5)	5 (50.0)
Mean ± SD	22.7 ± 12.5	19.2 ± 5.2
Bedriddeness rate, no. of patients (%)	0 (0.0)	0 (0.0)
Time from diagnosis to the first enrollment/incorporation, years		
Juvenile-onset (< 18 years)	1.75 ± 1.67	3.75 ± 2.92
Adult-onset (≥ 18 years)	0.80 ± 1.10	2.0 ± 1.41
All onset types	1.38 ± 1.50	3.4 ± 2.72
Plasma arginine concentration, µmol/L		
Prior to the onset of intravenous administration	63.1 ± 11.7	
Mean ± SD		NA
At 24 h after administration or at discontinuation		
Mean ± SD	163.5 ± 44.6	NA
Time from enrollment to the first ictus, days		
Median (95% CI)	397 (151.0)	NA
Number of ictuses assessed clinically, no. of patients (%)		
0	5 (38.5)	NA
1	2 (15.4)	7 (70.0)
2	2 (15.4)	2 (20.0)
≥ 3	4 (30.8)	1 (10.0)
Number of ictuses assessed by MRI, no. of patients (%)		
0	8 (61.5)	6 (60.0)
1	2 (15.4)	3 (30.0)
2	3 (23.1)	1 (10.0)
≥ 3	0 (0.0)	0 (0.0)
Annual increases in the JMDRS scores^c^		
Mean ± SD	1.98 ± 3.19	NA
JMDRS scores^c^ in sections 1 and 2		
0–10: no. of patients (%)	9 (69.2)	NA
11–20: no. of patients (%)	2 (15.4)	NA
21–30: no. of patients (%)	2 (15.4)	NA
≥ 31: no. of patients (%)	0 (0.0)	NA
All score ranges—mean ± SD	10.2 ± 8.48	NA

Values are expressed mean ± SD

*MELAS* mitochondrial myopathy, encephalopathy, lactic acidosis, and stroke-like episodes, *SD* standard deviation, *NA* not applicable, *CI* confidence interval, *MRI* magnetic resonance imaging, *JMDRS* Japanese mitochondrial disease rating scale

^a^Five patients each were enrolled in the clinical trials of oral and intravenous l-arginine, and four patients were enrolled in the clinical trial of intravenous l-arginine only. *N* represents the full analysis set

^b^Four patients, who had taken l-arginine other than the study drug prior to the onset of the present study, were enrolled in the clinical trial of intravenous l-arginine only

^c^Score range: 0–81

**Table 4:** Characteristics of pediatric and adult patients with interictal or acute MELAS who were followed up for 7 years after completion of the 2-year clinical trials of oral and intravenous l-arginine

Characteristics	Interictal MELAS (*N* = 13)^a,c^	Acute MELAS (*N* = 10)^a,d^
Sex		
Male, no. of patients (%)	7 (53.8)	8 (80.0)
Age, years		
< 18	0	0
≥ 18	11	8
Mean ± SD	30.6 ± 12.7	25.1 ± 5.1
Bedriddeness rate, no. of patients (%)	0 (0.0)	0 (0.0)
Mortality rate by onset type^e^, %		
Juvenile-onset (< 18 years)	2/8 (22.2)	1/8 (12.5)
Adult-onset (≥ 18 years)	0/5 (0.0)	1/2 (50.0)
All onset types	2/13 (15.4)	2/10 (20.0)
Duration of survival after diagnosis by onset type^f^, years		
Juvenile-onset (< 18 years)	10.8 ± 1.7	12.4 ± 3.3
Adult-onset (≥ 18 years)	9.8 ± 1.1	10.0
All onset types	10.4 ± 1.5	12.1 ± 3.1
Annual increases in the JMDRS scores^g,b^		
Juvenile (< 18 years)	1.39 ± 0.73	1.38 ± 0.84
Adult (≥ 18 years)	1.60 ± 0.63	–
All onset types	1.46 ± 0.78	1.38 ± 0.84
Progression of MELAS^g^, assessed using the JMDRS scores^b^		
0–10: no. of patients (%)	1 (10)	1 (14.3)
11–20: no. of patients (%)	4 (40)	2 (25.0)
21–30: no. of patients (%)	3 (30)	2 (25.0)
≥ 31: no. of patients (%)	2 (20)	2 (25.0)
All score ranges, mean ± SD	24.2 ± 11.7	28.7 ± 14.6
Progression of MELAS^g^, assessed using the NMDAS scores^h^		
0–20, no. of patients (%)	1 (10)	0 (0)
21–40, no. of patients (%)	4 (40)	4 (57.1)
41–60, no. of patients (%)	4 (40)	1 (14.3)
≥ 61: no. of patients (%)	1 (10)	2 (28.6)
All score ranges, mean ± SD	40.3 ± 22.5	48.3 ± 26.3

Values are expressed as mean ± SD

*MELAS*, mitochondrial myopathy, encephalopathy, lactic acidosis, and stroke-like episodes, *SD* standard deviation, *JMDRS* Japanese mitochondrial disease rating scale, *NMDAS* Newcastle mitochondrial disease adult scale

^a^Five patients with MELAS each were enrolled in the clinical trials of oral and intravenous l-arginine. Among 13 and 10 patients who completed the respective 2-year trials, two each died during the subsequent 7-year follow-up. No data on JMDRS and NMDAS were available for 1 “lost-to-follow-up but confirmed to be alive” patient each in the trials

^b^Score range: 0–81

^c^ *N* represents the full analysis set

^d^Five patients were enrolled in the clinical trial of intravenous l-arginine only

^e^The numbers of patients with interictal and acute MELAS were 13 and 10, respectively

^f^The numbers of patients with interictal and acute MELAS were 11 and 8, respectively, because 4 deaths: 1 each from renal failure and sudden death with respect to interictal MELAS, as well as 1 each from sudden death and renal and heart failure with respect to acute MELAS

^g^The numbers of patients with interictal and acute MELAS were 10 and 7, respectively, due to the absence of a qualified expert to treat MELAS

^h^Score range: 0–145

In the clinical trial of oral l-arginine, before-after analysis revealed no statistically significant difference in the MELAS stroke scale, mitochondrial disease severity scores, or migraine severity scores. Namely, l-arginine therapy failed to achieve the endpoints. Nevertheless, the *p* value was 0.0549 in changes in the MELAS stroke scale, indicating a tendency of oral l-arginine to improve symptoms. The interictal phases, which were assessed with the medians of the intervals between the day of first diagnosis and the day of first ictus and between 60 days prior to the day of first diagnosis and the day of first ictus, were longer after administration (*p* = 0.2500 and *p* = 0.0625, respectively; one-tailed test); thus, the interictal phases tended to be extended by oral l-arginine. Furthermore, the analysis of the ictus incidence ratios (IRRs: the pre- and postadministration incidences) disclosed that the IRRs were below 1 when using the ictuses that developed on the day of diagnosis or later [IRR 0.84; 95% confidence interval (CI) 0.52–1.36] and on 60 days prior to the day of diagnosis or later (IRR 0.57; 95% CI 0.37–0.86); thus, the IRRs of the ictuses tended to be lowered by oral l-arginine. The JMDRS scores in Sections 1 and 2 at baseline, the end of the 2-year clinical trials, and the end of the 7-year follow-up were 7.29 ± 8.77, 10.2 ± 8.48, and 24.2 ± 11.7, respectively (Tables 2, 3, 4).

**Fig. 2:**
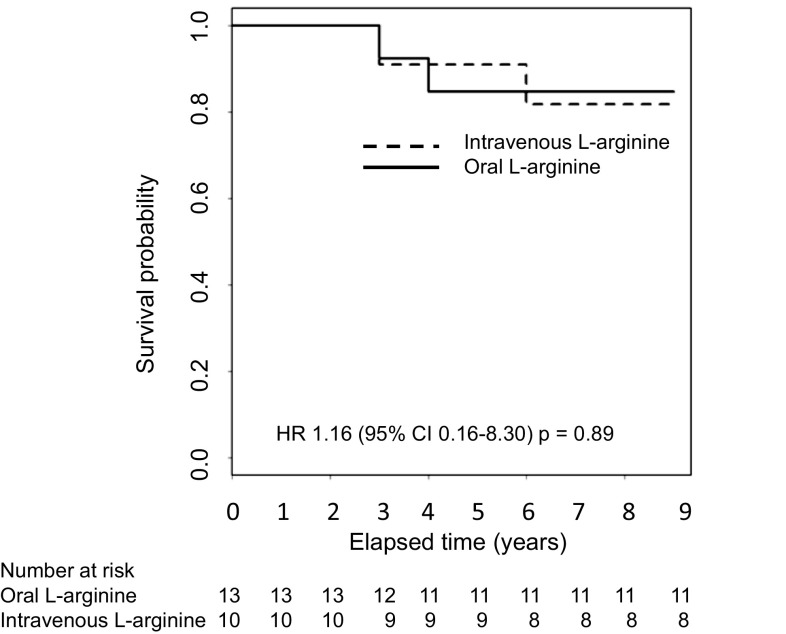
Kaplan–Meyer curve indicating the 9-year survival of patients who were treated with oral and intravenous l-arginine

Regarding the efficacy of intravenous l-arginine, furthermore, the improvement rates of headache and nausea/vomiting at 2 h after completion of the initial intravenous administration were 25.0% (2/8) and 50.0% (3/6) (*p* = 1.0000 each), respectively. Therefore, the statistical hypothesis that “the rate of improvement is greater than 30%” could not be tested for these co-primary endpoints. Nevertheless, the improvement rates of the co-primary endpoints, headache and nausea/vomiting, at 2, 6, 12, and 24 h after completion of the initial intravenous administration increased with time: for headache, 25.0% (2/8), 12.5% (1/8), 50.0% (4/8), and 62.5% (5/8), respectively; and for nausea/vomiting, 50.0% (3/6), 40.0% (2/5), 80.0% (4/5), and 80.0% (4/5), respectively. Furthermore, the improvement rates of four major symptoms increased with time: 100% (12/12) and 83.3% (10/12) according to the physician’s determinations and changes in the VAS scale. At the completion of the 2-year clinical trials, both the bedriddenness and mortality rates were 0% (Table 2) despite the progressively neurodegenerative and eventually life-threatening nature of MELAS. At the completion of 7-year follow-up, the bedriddenness rate remained to be 0%; in contrast, the mortality rates of patients with juvenile- or adult-onset MELAS were 22.2% and 0.0% in patients with interictal MELAS and were 12.5% and 50.0% in patients with acute MELAS (Table 4). The 9-year survival of patients who were treated with oral and intravenous l-arginine is shown in Fig. 2.

**Fig. 3:**
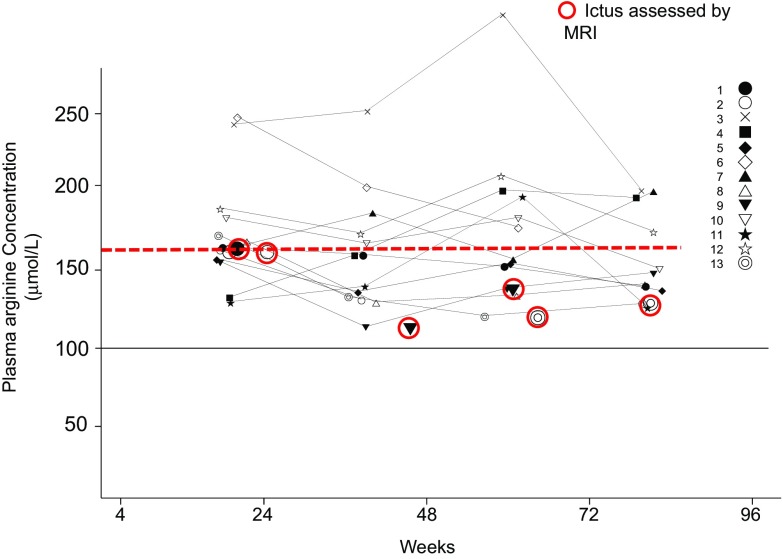
Relationships between plasma arginine concentrations and the ictuses of stroke-like episodes assessed by MRI. The red broken line indicates the maximal plasma arginine concentration (167 µmol/L) at which an ictus of stroke-like episodes developed. The black solid line indicates the plasma arginine concentration that is required for the normalization of endothelial function. *MRI* magnetic resonance imaging

Plasma arginine concentrations at baseline and week 96 in the clinical trial of oral l-arginine were 65.74 ± 16.74 μmol/L and 162.36 ± 45.23 μmol/L, respectively, in 13 patients each who had interictal MELAS. Plasma arginine concentrations prior to the onset of intravenous administration and at 2 h after completion of the initial intravenous administration or discontinuation were 89.81 ± 76.25 μmol/L and 143.44 ± 122.78 μmol/L, respectively, in 10 patients with acute MELAS each in the clinical trial of intravenous l-arginine. In a total of 13 patients with interictal MELAS, the relationships between plasma arginine concentrations at weeks 4, 24, 48, 72, and 96 with the ictuses of stroke-like episodes assessed by MRI were examined. Consequently, the ictuses developed at a plasma arginine concentration of 167 µmol/L or below (Fig. 3). Plasma arginine concentrations at 2 h after oral administration were maintained at 100 μml/L or higher. The median concentrations (range) prior to and at 24 h after completion of the initial intravenous administration were 51.15 (28.3–224.9) µmol/L and 111.30 (31.2–520.2) µmol/L, respectively.

Oral l-arginine was well tolerated, and 13 of 15 patients completed the 2-year clinical trial. All of 15 patients receiving oral l-arginine developed stroke-like episodes originating from MELAS that were adjudicated to be incidental, not to be related with oral l-arginine, and mild to moderate in severity. Nasopharyngitis (66.7%, 10/15) was the most common AE. The following seven episodes of severe AEs developed: drug hypersensitivity, increased aspartate aminotransferase, increased alanine aminotransferase, increased creatinine phosphokinase, metabolic acidosis, arrhythmias, and volvulus. All these patients recovered or became alleviated due to the discontinuation or withdrawal of l-arginine. Therefore, oral l-arginine was administrable for a long time through careful monitoring of laboratory abnormalities.

On the other hand, 6 (42.9%) among a total of 10 patients who received intravenous l-arginine developed AEs. Fever was the most common AE in 5 (50.0%) of 10 patients. Decreased hematocrit and hemoglobinuria were found in three patients each (30.0%), and patients recovered without treatment. Six episodes of moderate AEs developed: four of fever and one each of epilepsy and bleeding at the injection site; causality with intravenous l-arginine was denied. One episode of severe seizures developed in a patient who had been prone to develop seizures since before the trial. Laboratory data and vital signs did not show any changes of clinical relevance.

## Discussion

The present research has integrated the pooled data from two small-sized clinical trials of oral and l-arginine in Japanese patients with MELAS that did not adopt the “placebo-controlled design”, because (1) strong ethical concerns were elicited by MELAS a miserable mitochondrial disease that could cause irreversible brain damage so severe as leading to bedriddenness after one ictus only in patients who would have received placebo and (2) impossibility of obtaining informed consent from candidate patients to accept a 50% probability of receiving placebo despite such a horrible feature of the disease under the current universal health insurance system in Japan, based on which the institutional review board at the participating sites necessarily led to abandon the initially designed randomized placebo-controlled trial. Furthermore, the trials failed to demonstrate statistically significant differences in the primary endpoints. Nevertheless, our research provides physicians with the following insights of paramount clinical relevance: (1) maintaining plasma arginine concentration ≥ 168 µmol/L may prevent the ictuses through the putative pathophysiologic mechanism optimal normalization of endothelial dysfunction [11]; (2) the bedriddenness rate was 0% at the completion of both the 2-year clinical trials and the 7-year follow-up, in marked contrast to 5.2% in the 5-year cohort study [6]; (3) the mortality rates 0% in five patients with adult-onset, interictal MELAS who continued receiving oral l-arginine during the interictal phase and were appropriately treated with intravenous l-arginine during the acute phase and 50.0% in two patients with adult-onset, acute MELAS who were treated with intravenous l-arginine but were not treated with oral l-arginine during the interictal phase infer that this therapeutic regimen is appropriately rationalized to prevent the progression and fatal outcome of MELAS; (4) sudden death occurred in two patients with juvenile-onset MELAS, one each in the clinical trials of oral and intravenous l-arginine, implying the eventually life-threatening nature of MELAS; (5) MELAS was well controlled in the 2-year clinical trials as indicated by a little change in the JMDRS scores; and (6) this therapeutic regimen alone was not sufficient to stem disease progression during the 7-year follow-up in real-world clinical practice, where the clinical control of patients became less strict, as evidenced by greater increases in the JMDRS scores compared to the scores’ changes found at the end of the 2-year clinical trials, i.e., the invariably progressive nature of MELAS was confirmed and disease progression might have been furthered by less rigorous clinical control. However, our results afford clinical evidence on the therapeutic regimen of l-arginine therapy that is enough to satisfy the “unmet desire [18]” of pediatricians and neurologists around the world who are daily dedicated to the treatment of patients with MELAS.

Moreover, the abovementioned insights have the following medical implications for the routine care of patients with MELAS: (1) these patients can be well controlled and disease progression may be retarded when this therapeutic regimen is conducted strictly on an as-needed basis; (2) due to the progressively neurodegenerative and eventually life-threatening nature of MELAS, the regimen may require further therapeutic contrivances (e.g., a medication more strictly tailored to individual patients and the regular monitoring of plasma arginine concentrations) in an attempt to prevent or retard disease progression for years; and (3) clinicians who treat patients with MELAS are strongly encouraged to implement the regimen as strictly as for clinical trials.

l-Arginine is a precursor for nitric oxide (NO) synthesis, and its oral or intravenous administration at relatively large doses has been shown to enhance the formation of NO in individuals with impaired endothelial function [19]. The impaired formation or dysfunction of NO in the vasculature is an important pathogenic factor in the development of vasculopathies [20]. On the other hand, asymmetrical dimethylarginine (ADMA)—an endogenous competitive inhibitor of NO synthase [19, 21] and a risk factor for ischemic heart disease [13], atherosclerosis [19, 21], and endothelial dysfunction [9–14, 22–28]—may determine an individual’s response to l-arginine supplementation [20]; its accumulation impairs the formation of NO in vascular diseases [25]. Patients with MELAS invariably show endothelial dysfunction [10–14, 21, 22, 25, 26] that is caused by the impairment of endothelium-dependent, NO-mediated vasodilatation in which l-arginine plays an important role [10–14, 24, 26]. Low plasma arginine concentrations and relatively high ADMA concentrations in patients with MELAS may predispose the ictus of stroke-like episodes [10, 12, 24, 26], and endothelial dysfunction can be reversed by exogenous l-arginine [10–12, 26]. Hence, clinicopathologic evidence underpins l-arginine therapy for patients with MELAS [10–14, 27–30].

All patients receiving oral l-arginine developed AEs (e.g., nasopharyngitis, drug hypersensitivity, and increased lactate dehydrogenase). However, all AEs found during oral or intravenous l-arginine were transient and manageable clinically without requiring medications in particular, and any severe concerns about the safety of these formulations were not elicited at all. These are fully in line with the fact that intravenous l-arginine is generally used, without any severe reported AEs, the screening for growth hormone insufficiency around the world.

Little long-term clinical study is available that has examined the disease severity and progression of mitochondrial diseases that involve the most common variant m.3243 A>G in the mt-rRNA^Leu^. In 2018, a long-term cohort study in the UK [31] was published that had recruited 242 adult m.3243A>G carriers between 2005 and 2017 to determine which putative prognostic factors are most associated with m.3243A>G-related mitochondrial disease burden and progression. Consequently, increasing age as well as interactions between age and increasing age-adjusted blood mitochondrial DNA heteroplasmy level were significantly positively associated with disease progression (*p* < 0.001), although interindividual variation was extremely high. In consideration of the chronically progressive and eventual fatal clinical course of MELAS, clinicians who treat patients with mitochondria diseases are advocated to use patient age and age-adjusted blood m.3243A>G mutation load as indicators for disease burden and progression.

The present research has several limitations. First, the number of patients with MELAS was small—a fact that reduces the statistical impact of the present research. However, one can never make a simple comparison with clinical trials on other diseases that affect a much greater number of individuals. Therefore, due regard should be given to the fact that MELAS a major clinicopathologic type of mitochondrial diseases is an orphan disease (estimated prevalence rate of 10–15 cases per 100,000 persons) that was designated to be one of intractable disorders by the Japanese government in 2009. We had barely identified among 233 patients with MELAS in whole Japan 96 patients who could complete a 5-year, nationwide, multicenter, prospective cohort study [6] the only nationwide epidemiologic study on this patient group available in the world that revealed the natural course of patients with MELAS (time from diagnosis to death: 7.3 ± 5.0 years; mortality rate: 20.8%). In the present clinical research, 15 and 10 patients among these 96 patients constituted the clinically valuable groups of patients who underwent treatment with oral and intravenous l-arginine, respectively. In real-world clinical practice, the number of patients with an orphan disease like MELAS in long-term clinical research would be inevitably limited when considering its epidemiologic profiles. Second, the study design was not “placebo-controlled” due to strong ethical concerns as described previously. Hence, selection, verification, or incorporation bias cannot be ruled out. Third, the primary endpoints of the clinical trial of l-arginine could not be achieved. We speculate that the extended window for intravenous administration from “3 h” in our previous clinical study [9] to “6 h” in the present clinical trial might have disadvantageously attenuated the pharmacological effects of intravenous l-arginine. All these methodological and statistical issues may restrict the unobjectionable acceptance of our data. Nevertheless, our research is therapeutically unique in the following achievements: (a) specification of the plasma arginine concentration (trough level: 168 µmol/L) at which l-arginine is of potential reference value for preventing an ictus that may cause irreversible brain damage, or bedriddeness in the worst case—however, its validation by the further accumulation of clinical evidence is required; (b) presentation of “0%” in the bedriddenness rate during the strict systematic administration of oral and intravenous l-arginine in the 2-year clinical trials; and (c) the obvious suppression and modest retardation of disease progression at the ends of 2-year clinical trials and 7-year follow-up, respectively.

In conclusion, the present research underpins the systematic administration of oral and intravenous l-arginine. Therefore, this regimen may be therapeutically beneficial and clinically useful for patients with MELAS. A placebo-controlled study of oral and intravenous l-arginine in patients with MELAS will provide clinical evidence to strengthen the findings of the present research.
